# Calmodulin-dependent KCNE4 dimerization controls membrane targeting

**DOI:** 10.1038/s41598-021-93562-5

**Published:** 2021-07-07

**Authors:** Sara R. Roig, Laura Solé, Silvia Cassinelli, Magalí Colomer-Molera, Daniel Sastre, Clara Serrano-Novillo, Antonio Serrano-Albarrás, M. Pilar Lillo, Michael M. Tamkun, Antonio Felipe

**Affiliations:** 1 Molecular Physiology Laboratory, Dpt. de Bioquímica I Biomedicina Molecular, Institut de Biomedicina (IBUB), Universitat de Barcelona, Avda. Diagonal 643, 08028 Barcelona, Spain; 2 Imaging Core Facility, Biozentrum, University of Basel, 4056 Basel, Switzerland; 3 Department of Biomedical Sciences, Colorado State University, Fort Collins, CO 80523 USA; 4 Instituto de Química Física Rocasolano, CSIC, 28006 Madrid, Spain

**Keywords:** Cell biology, Neuroscience

## Abstract

The voltage-dependent potassium channel Kv1.3 participates in the immune response. Kv1.3 is essential in different cellular functions, such as proliferation, activation and apoptosis. Because aberrant expression of Kv1.3 is linked to autoimmune diseases, fine-tuning its function is crucial for leukocyte physiology. Regulatory KCNE subunits are expressed in the immune system, and KCNE4 specifically tightly regulates Kv1.3. KCNE4 modulates Kv1.3 currents slowing activation, accelerating inactivation and retaining the channel at the endoplasmic reticulum (ER), thereby altering its membrane localization. In addition, KCNE4 genomic variants are associated with immune pathologies. Therefore, an in-depth knowledge of KCNE4 function is extremely relevant for understanding immune system physiology. We demonstrate that KCNE4 dimerizes, which is unique among KCNE regulatory peptide family members. Furthermore, the juxtamembrane tetraleucine carboxyl-terminal domain of KCNE4 is a structural platform in which Kv1.3, Ca^2+^/calmodulin (CaM) and dimerizing KCNE4 compete for multiple interaction partners. CaM-dependent KCNE4 dimerization controls KCNE4 membrane targeting and modulates its interaction with Kv1.3. KCNE4, which is highly retained at the ER, contains an important ER retention motif near the tetraleucine motif. Upon escaping the ER in a CaM-dependent pattern, KCNE4 follows a COP-II-dependent forward trafficking mechanism. Therefore, CaM, an essential signaling molecule that controls the dimerization and membrane targeting of KCNE4, modulates the KCNE4-dependent regulation of Kv1.3, which in turn fine-tunes leukocyte physiology.

## Introduction

Voltage-dependent potassium (Kv) channels participate in the resting membrane potential and repolarization of excitable cells^1^. Furthermore, Kv channels mediate additional cellular functions, such as control of cell cycle progression, cell activation and apoptosis^2^. Kv1.3 is mainly expressed in the nervous and immune systems. Leukocytes present a limited repertoire of potassium channels, and Kv1.3 is essential during the immune system response^3,4^. T lymphocyte activation triggers the translocation of the channel to the immunological synapse between T lymphocytes and antigen-presenting cells, maintaining the driving force for sustained calcium signaling^5^.

Regulatory subunits modulate the function, trafficking and subcellular localization of Kv channels^6^. The KCNE family contains five single-span membrane auxiliary peptides (KCNE1-5) that modulate potassium channels^7^. KCNEs are present in the immune system, and their expression is regulated by several insults, highlighting the importance of this peptide family in leukocytes^8,9^. KCNE4, the largest member of the family, triggers dominant-negative effects on Kv channels. For instance, this subunit decreases Kv1.1, Kv1.3, Kv2.1 and Kv7.1 potassium current^10–12^. KCNE4 inhibits Kv7.1 current, not by altering the surface abundance of the channel but by altering the channel localization in lipid raft microdomains^13,14^. Functional interactions between KCNE4 and Kv7.1 require the presence of calmodulin (CaM). In fact, KCNE4 interacts with Ca^2+^/CaM through a tetraleucine domain in the juxtamembrane of the KCNE4 C-terminus^15^.

Evidence demonstrates that alterations in Kv1.3 and KCNE4 are associated with immune system pathologies^16–18^. KCNE4, tightly regulated in leukocytes, inhibits Kv1.3 function by hindering the surface expression and lipid raft localization of the channel^19^. Kv1.3 and KCNE4 interact via the C-terminal domains of both proteins^20,21^. While the implicated molecular determinants of Kv1.3 are not evident, the tetraleucine domain of KCNE4 clearly participates in this interaction^20,21^. The tetraleucine motif hub forms a competing mechanism that directs the KCNE4 association with either CaM or Kv1.3, tuning the membrane targeting of the channel. The fact that several dileucine-rich domains participate in protein–protein interactions and that the tetraleucine signature of KCNE4 facilitates the association with CaM and Kv1.3 points to whether this element could be involved in further interactions regulated by CaM. In fact, KCNE4 does not inhibit Kv7.1 by impairing the association to CaM^15^. Furthermore, CaM interaction improves the surface targeting of Kv7.2^22^. Therefore, the evidence suggests that the CaM interaction, and hence the molecular determinants involved, is an important intracellular signaling mechanism of ion channel function.

We show in this work that the tetraleucine motif in the juxtamembrane region of the KCNE4 C-terminus functions as a multiple platform for subunit assembly and protein interaction. In contrast with other members of the KCNE family, KCNE4 forms stable dimers. The tetraleucine domain, unique to KCNE4, mediates this dimerization. The association of KCNE4 with CaM, as well as with Kv1.3, disrupts the dimer and facilitates the cell surface expression of KCNE4. CaM fine-tunes the association capacity of KCNE4 with Kv1.3, thereby modulating channel-dependent immune responses. Our data are of physiological relevance because CaM, a very important intracellular signaling molecule, controls Kv1.3-dependent KCNE4-related immunological events.

## Results

### KCNE4, broadly expressed in mammalian tissues, specifically inhibits Kv1.3

**Figure 1:**
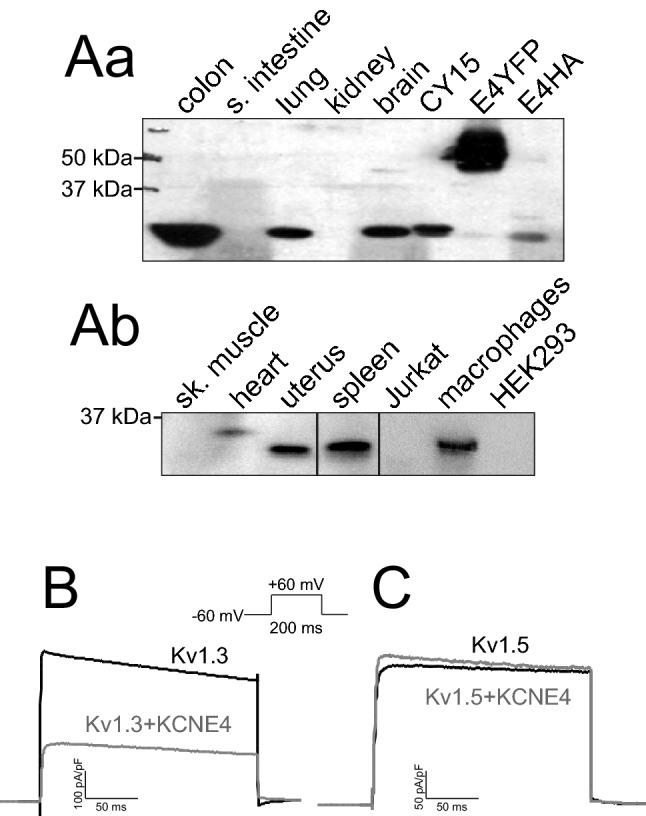
KCNE4, which specifically regulates Kv1.3, is differentially expressed in tissues and leukocytes. (**A**) Protein expression of KCNE4 in different rat tissues and cell lines. HEK-293 cells were used as negative control, and HEK cells transfected with KCNE4-YFP (E4YFP) and KCNE4-HA (E4HA) were used as positive controls. Different western blots with the same KCNE4-YFP and KCNE4-HA controls from different origins were merged for better visualization. Fifty micrograms of protein was loaded in each lane, and filters were immunoblotted with anti-KCNE4 antibody. Differential β-actin expression among tissues and cell lines (not shown) invalidated this protein as a control. Therefore, a KCNE4 abundance comparison among samples should not be considered. The results must be considered as qualitative data. Representative cropped blots, clearly separated by vertical black lines, are shown only for qualitative purposes. (**B**, **C**) Representative patch-clamp recordings of HEK-293 cells transfected with Kv1.3 and Kv1.5 in the absence or presence (+ KCNE4) of KCNE4. Cells were hold at -60 mV and voltage-dependent K^+^ currents were elicited by applying 200 ms depolarizing pulses to + 60 mV. (B) Kv1.3 + /- KCNE4. (**C**) Kv1.5 + /- KCNE4. Gray lines, presence of KCNE4 (+ KCNE4); black lines, absence of KCNE4. Data analysis was performed using FitMaster (HEKA) and SigmaPlot 10.0 software (Systat Software).

In addition to cardiac action potentials and synaptic transmission, Kv channels participate in many physiological functions throughout the body. The expression of these proteins is not limited to excitable cells, and tight modulation by regulatory subunits is essential to achieve a plethora of actions. The association with regulatory peptides adds complexity to the channel architecture, further increasing their function. In this scenario, KCNE4 modulates many Kv channels, and KCNE4 mRNA expression is ubiquitous^10,11,23^. However, no protein data are available. We showed that KCNE4 was differentially expressed in several rat tissues and leukocytes (Fig. 1A). As previously mentioned^10,11^, we confirmed the expression of KCNE4 in the uterus, which is not surprising because the uterus exhibits ion channel proteins, such as KCNE1 or Nav2.3 ^24,25^. KCNE4 was also present in some epithelium-like tissues, such as the colon and lung (Fig. 1Aa), where the movement of ions and water is achieved by multiple ion channels. The presence of KCNE4 in the brain and heart is in accordance with several Kv proteins, such as Kv1.1, Kv1.3, Kv2.1, and Kv7.1, which are targets of this regulatory peptide^23^. Within the immune system, in contrast to Jurkat T lymphocytes, KCNE4 expression in spleen and mononuclear phagocytes, such as CY15 dendritic cells (Fig. 1Aa) and bone marrow-derived macrophages (Fig. 1Ab), was notable. Although KCNE4 is widely expressed in the immune system, KCNE4 regulation is unique. Leukocytes express both Kv1.3 and Kv1.5 to govern cell responses^3,26^, but KCNE4 selectively regulates only Kv1.3 when expressed in HEK-293 cells (Fig. 1B, C).

### KCNE peptides exhibit important structural motifs in the juxtamembrane region of the C-terminus

**Figure 2:**
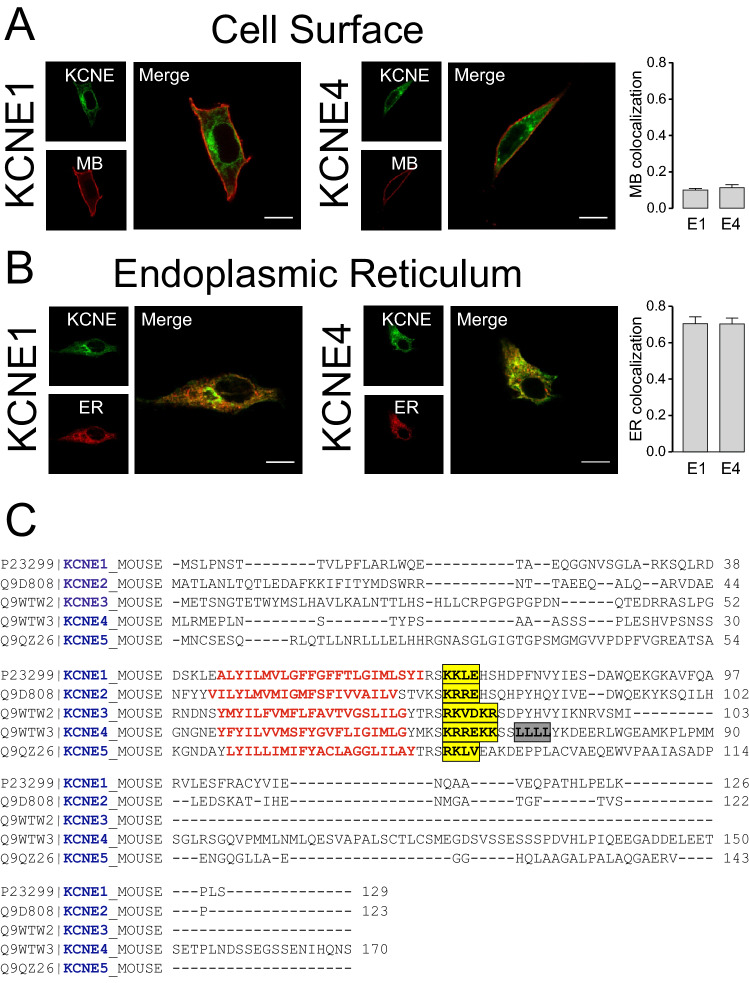
KCNE1 and KCNE4 exhibit notable endoplasmic reticulum retention. Representative confocal images of HEK-293 cells transfected with KCNE1 and KCNE4. (**A**) KCNE-transfected cells were cotransfected with a plasma membrane marker (MB). (**B**) KCNE-transfected HEK-293 cells were cotransfected with an endoplasmic reticulum marker (ER). Right panels, KCNE1; center panels, KCNE4; left panels, histograms showing the colocalization between KCNEs (E1 and E4) and markers (MB and ER) based on Mander’s coefficient analysis. The values represent the mean of > 40 cells. Green, KCNEs; red, cell markers; yellow, merged image. Bars represent 10 μm. (**C**) Sequence alignment of murine KCNE peptides. KCNE1 (UniProtKB: P23299); KCNE2 (UniProtKB: Q9D808); KCNE3 (UniProtKB: Q9WTW2); KCNE4 (UniProtKB: Q9WTW3); KCNE5 (UniProtKB: Q9QZ26). Mouse sequences are shown because murine isoforms were used throughout the study, and the results obtained were similar to the results with human isoforms. Transmembrane segments are highlighted in red. The ER-retention motifs are boxed in yellow. The specific tetraleucine motif identified in KCNE4 is colored gray.

KCNE regulatory peptides, sharing a notable ER retention, cause a differential effect on the plasma membrane targeting of Kv channels^13,14^. Thus, while KCNE1 interaction mostly improves Kv channel membrane targeting^27^, KCNE4 transfers to the Kv1.3 complex ER retention motifs crucial for the control of the surface expression of the channel^20,21^. Because of the physiological relevance of these effects on the immune response, we characterized the intracellular retention mechanisms of KCNE4 by using KCNE1, the most documented KCNE subunit, as a reference. The membrane targeting of KCNE1 was low and similar to that observed with KCNE4 (Fig. 2A), and both KCNEs markedly colocalized with the ER (Fig. 2B). Our data further support and extend the evidence suggesting that KCNEs share ER retention motifs in their structure. A sequence alignment of their primary structures clearly shows basic clusters canonically associated with ER retention located in the juxtamembrane region of the KCNE C-terminus (Fig. 2C). In this context, KCNE3 and KCNE4 motifs contained larger motifs, which could suggest a major capacity of transfer intracellular retention to the associated Kv complex. Interestingly, KCNE4 is unique within the KCNE family because it presents a tetraleucine motif (L69-72) known to participate in the CaM-dependent modulation of channels, such as Kv7.1 and Kv1.3^15,20^.

### KCNE4 dimerization

**Figure 3:**
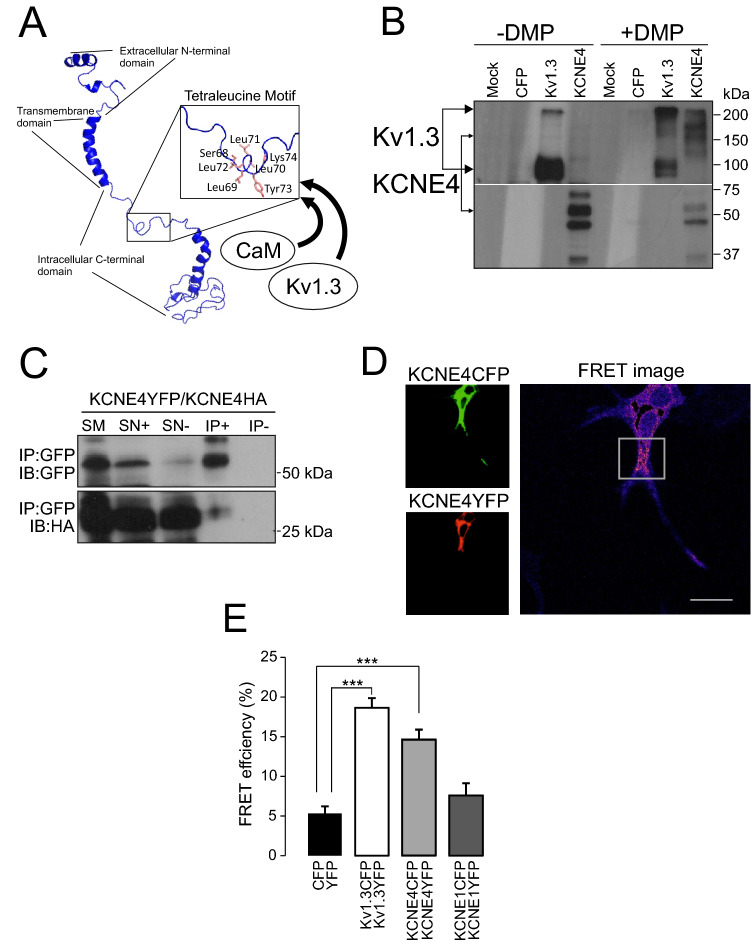
KCNE4 homo-oligomerization. HEK-293 cells were transfected with different KCNE4-tagged peptides, and their homo-oligomeric formation ability was studied. (**A**) Molecular simulation of KCNE4 using the KCNE1 template (PDB code 2K21) as described in the Materials and Methods. The inset highlights the tetraleucine signature (L69-72A) where CaM and Kv1.3 associate. (**B)** Oligomerization of KCNE4. Cell lysates were incubated in the absence ( −) or presence ( +) of DMP (dimethyl pimelimidate). Filters were immunobloted against CFP (Kv1.3-CFP, KCNE4-CFP). Lower Kv1.3 arrow, Kv1.3 monomers. Upper Kv1.3 arrow, Kv1.3 tetramers. Lower KCNE4 arrow, KCNE4 monomers. Upper KCNE4 arrow, KCNE4 oligomers. Mock- and CFP-transfected cells were used as negative controls. (**C**) HEK-293 cells were cotransfected with KCNE4-YFP and KCNE4-HA. Immunoprecipitation was performed for KCNE4-YFP (IP: GFP). Top panel: Immunoblot (IB) against GFP. Bottom panel: immunoblot (IB) against HA. SM: starting material. SN + , supernatant in the presence of antibody. SN-, supernatant in the absence of antibody. IP + , Immunoprecipitation in the presence of the anti-GFP antibody. IP- Immunoprecipitation in the absence of the anti-GFP antibody. (**D**, **E**) HEK-293 cells were transfected with Kv1.3-, KCNE1- and KCNE4-tagged (CFP/YFP) proteins. Homo-oligomerization of Kv1.3 and KCNE4, but not KCNE1, as analyzed by the FRET acceptor-photobleaching technique. (**D**) Green, representative image of a KCNE4-CFP donor. Red, representative image of a KCNE4-YFP acceptor. FRET image, white square highlights a ROI showing FRET image obtained after photobleaching. Bars represent 10 μm. (**E**) Quantification of the FRET efficiency (%). CFP/YFP, negative control; Kv1.3CFP/Kv1.3YFP, positive control. ***, *p* < 0.001 vs CFP/YFP (Student’s *t*-test). The values represent the mean ± SEM of 22 (CFP/YFP), 33 (Kv1.3CFP/Kv1.3YFP), 28 (KCNE4CFP/KCNE4YFP) and 25 (KCNE1CFP/KCNE1YFP) cells.

Because dileucine motifs are critical for multiple protein–protein interactions, we wondered whether the tetraleucine signature mediates homo-oligomerizations of KCNE4. If it does, then this subunit is unique within the KCNE family, conferring distinctive structural features to this peptide that may fine-tune its Kv1.3-specific physiological functions. The molecular modeling of KCNE4, based on the KCNE1 structure (PBD ID: 2K21)^28^, suggested that the tetraleucine motif, containing four consecutive hydrophobic residues, forms an accessible loop for different protein–protein interactions, e.g. Kv1.3 and CaM (Fig. 3A). This model is highly reliable because, although it is based on the KCNE1 structure^28^, it is quite similar to that obtained for KCNE3 by cryo-EM^29^. Protein analysis in the presence of the DMP cross-linking reagent revealed that KCNE4 and Kv1.3 form oligomeric structures, which indicates several protein–protein interactions (Fig. 3B). Thus, the tetrameric Kv1.3-YFP complex forms large molecules. Similarly, KCNE4 exhibited large forms, which indicate multimeric complexes detectable only with DMP. The formation of these structures was confirmed by coimmunoprecipitation showing that KCNE4-YFP and KCNE4-HA interact (Fig. 3C) and FRET studies of KCNE4-YFP and KCNE-4CFP (Fig. 3D and E). This result was specific for KCNE4 because KCNE1 showed no positive FRET values (Fig. 3E).

**Figure 4:**
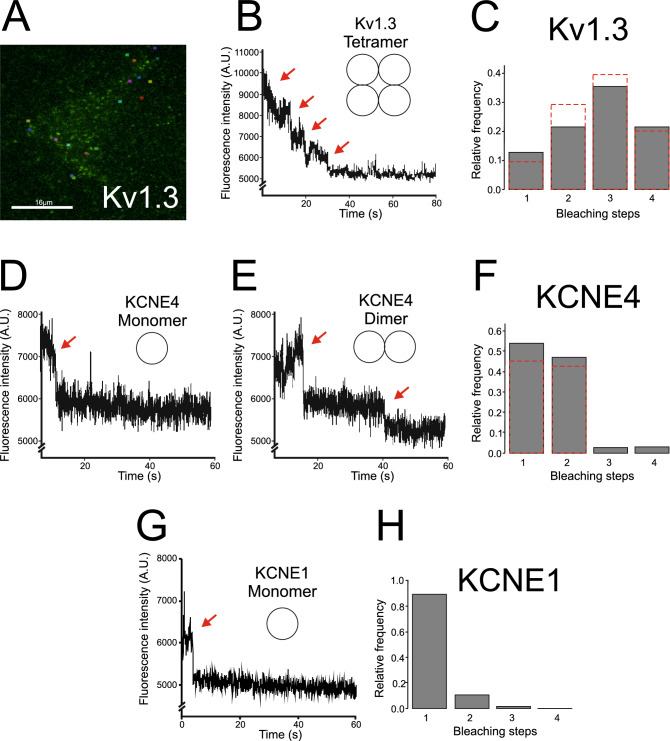
GFP bleaching steps from HEK-293 cells transfected with Kv1.3, KCNE4 and KCNE1. (**A**–**C**) Cells were transfected with Kv1.3 loopBADGFP. (**A**) Representative snapshot from the TIRFM video (488 nm laser). Colored ROIs (6 × 6 pixels) indicate representative analyzed unmoving spots. Scale bar represents 16 μm. (**B**) Representative graph of bleaching steps from different spots that were analyzed. Red arrows point to 4 bleaching steps. (**C**) Relative frequency of 1–4 bleaching events counted per spot. Gray bars correspond to the experimental frequency observed. Red dashed lines correspond to the theoretical distribution of the bleaching steps with *p* = 0.67. (**D–F**) GFP bleaching steps with HEK-293 cells transfected with KCNE4 loopBADGFP. (**D**, **E**) Representative graphs of the bleaching steps from different KCNE4 spots that were analyzed. (**D**) Red arrows point to one bleaching step, indicating a KCNE4 monomer. (**E**) Two bleaching steps demonstrating KCNE4 dimers. (**F**) Relative frequency of 1–4 bleaching events counted per spot. Gray bars correspond to the experimental frequency observed. Red dashed lines correspond to the theoretical distribution of bleaching steps showing a dimer. (**G**, **H**) GFP bleaching steps from HEK-293 cells transfected with KCNE1 loopBADGFP. (**G**) Representative graph showing one bleaching step suggesting KCNE1 monomers. (**H**) Relative frequency of 1–4 bleaching events counted per spot. Gray bars correspond to the experimental frequency observed, demonstrating a KCNE1 monomeric structure.

To characterize the KCNE4 oligomers further, we performed a TIRF-derived single bleaching step assay^30^. Kv1.3-loopBAD-GFP, KCNE4-loopBAD-GFP and KCNE1-loopBAD-GFP were expressed in HEK 293 cells, and GFP fluorescent immobile spots were monitored (Fig. 4). Kv1.3 was used as the control (Fig. 4A–C). Experimental bleaching steps in selected ROIs demonstrated the canonical Kv1.3 tetrameric architecture (Fig. 4B). We calculated the expected distribution of the tetrameric channels with different p values (probability of fluorescing GFP), and the best fit was obtained with a GFP folding efficiency of 67% (*p* = 0.67) (Fig. 4C), indicating a tetrameric architecture as previously described^30–32^. However, KCNE4 mostly exhibited two unique possible structures (Fig. 4D–F). That is, monomeric and dimeric forms of KCNE4 were detected (Fig. 4D and E). The best fit for the expected distribution and the experimental data revealed that KCNE4 is present in monomeric and dimeric forms (Fig. 4F). In contrast, using the same approach, we found that KCNE1 was present only in monomeric form (Fig. 4G, H), as previously demonstrated^33,34^.

### The juxtamembrane tetraleucine motif facilitates CaM-dependent protein–protein interactions and membrane targeting of KCNE4

**Figure 5:**
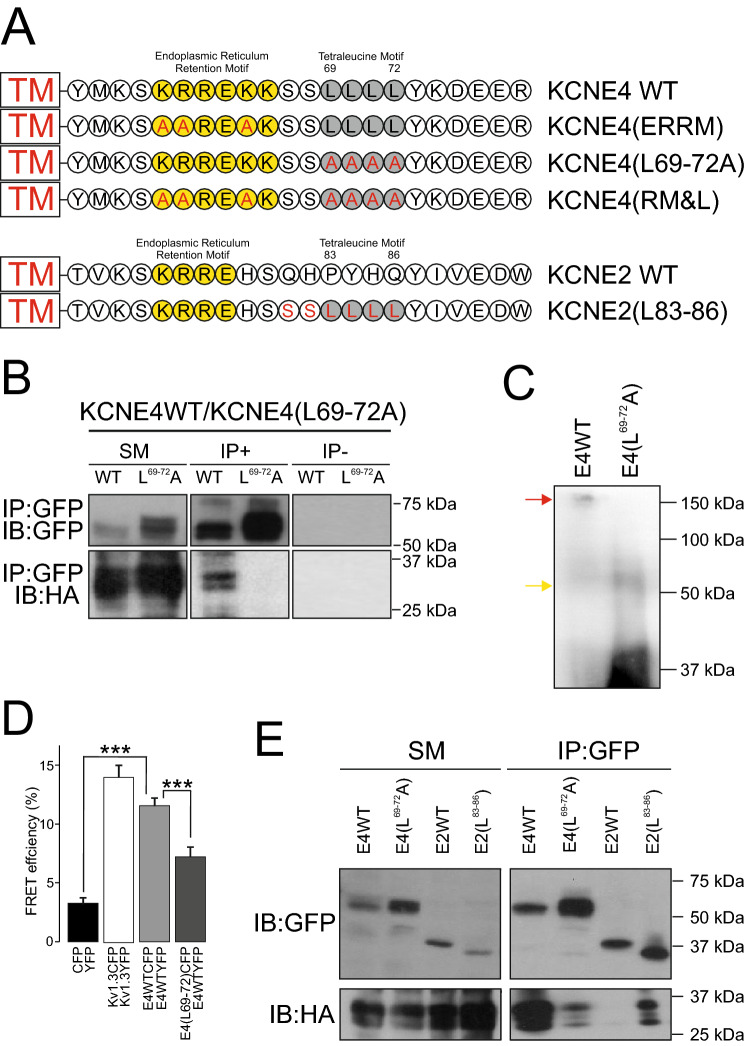
The juxtamembrane tetraleucine motif is critical for KCNE4 dimerization. (**A**) Representative cartoon showing the sequence alignment of the juxtamembrane segment of KCNE4 and KCNE2 and the comparison with the introduced mutations. The transmembrane domain (TM) is shown in a box in red. The KCNE ERRMs are in yellow. The tetraleucine signature is colored gray. Amino acid substitutions are highlighted in red. For KCNE4 (ERRM), the ERRM of KCNE4 was disrupted with alanine residues (in red). For KCNE4 (L69-72A), the tetraleucine motif (gray) was also mutated to alanine (in red). For KCNE4(RM&L), both the ERRM and the tetraleucine L69-72A motifs were disrupted by alanine substitutions. The tetraleucine motif of KCNE4 was introduced in the same location in KCNE2 (KCNE2(L83-86)). (B-D) HEK-293 cells were transfected with KCNE4CFP, KCNE2-HA, Kv1.3-(CFP/YFP) and different KCNE mutants. (**B**) Coimmunoprecipitation assay against KCNE4-CFP and KCNE4(L69-72A)CFP in the presence of KCNE4-HA (IP:GFP). Top panel: Immunoblot against CFP (IB:GFP). Bottom panel: immunoblot against HA (IB:HA). SM: starting material. IP + : Immunoprecipitation in the presence of the anti-GFP antibody. IP-: Immunoprecipitation in the absence of the anti-GFP antibody. KCNE4-HA coimmunoprecipitates with KCNE4CFP (KCNE4WT) but not with KCNE4(L69-72A)CFP. Representative cropped blots are clearly separated by vertical white lines. (**C**) Non-denaturing SDS-PAGE. HEK 293 cells were transfected with KCNE4WT-CFP and KCNE4(L69-72A)CFP and cell lysates immunoblotted against GFP. Note that while KCNE4WT-CFP exhibited large molecular mass forms (red arrow), KCNE4(L69-72A)CFP only appeared as monomers (yellow arrow). (**D**) Quantification of the FRET efficiency of KCNE4 dimerization. Cells were cotransfected with CFP/YFP (negative controls), Kv1.3CFP/Kv1.3-YFP (positive control), KCNE4WT (CFP/YFP) and KCNE4WT-YFP and KCNE4(L69-72A)CFP. The values represent the mean ± SE of n > 25 cells. ***p < 0.001 Student’s *t*-test. (E) HEK-293 cells were cotransfected with KCNE4WT-HA/ KCNE4(L69-72A)CFP and KCNE4WT-HA and either KCNE2WT-CFP or KCNE2(L83-86)-CFP. Top panels: Immunoblot against CFP (IB: GFP). Bottom panel: immunoblot against HA (IB: HA). SM: starting material. IP: GFP: Immunoprecipitation in the presence of the anti-GFP antibody. KCNE4-HA coimmunoprecipitates with KCNE2(L83-86)-CFP but not with KCNE2WT-CFP.

The tetraleucine motif of KCNE4 is an interacting platform that defines the CaM modulation of the KCNE4-dependent regulation of Kv1.3 and Kv7.1^15,20^. In this context, we wanted to know whether this unique motif among KCNE peptides facilitates KCNE4 dimerization. To examine this possibility, we generated several KCNE4 mutants that disrupted the tetraleucine motif either alone (KCNE4(L69-72A)) or in combination with ERRM signaling (KCNE4(RM&L)). KCNE2, which neither contains tetraleucine motifs nor associates with Kv1.3, has been widely characterized^20^. Therefore, a KCNE2 mutant (KCNE2(L83-86)) with an embedded tetraleucine motif was also used (Fig. 5A). In contrast to WT KCNE4, disruption of the tetraleucine motif (KCNE4(L69-72A)) impaired KCNE4 dimerization, as observed by coimmunoprecipitation (Fig. 5B), non-denaturing polyacrylamide gel electrophoresis (Fig. 5C) and further supported by FRET assays (Fig. 5D). In addition, coimmunoprecipitation experiments with the KCNE2(L83-86) mutant showed that the addition of the leucine cluster to KCNE2 triggered the dimerization of KCNE2 with KCNE4 (Fig. 5E).

**Figure 6:**
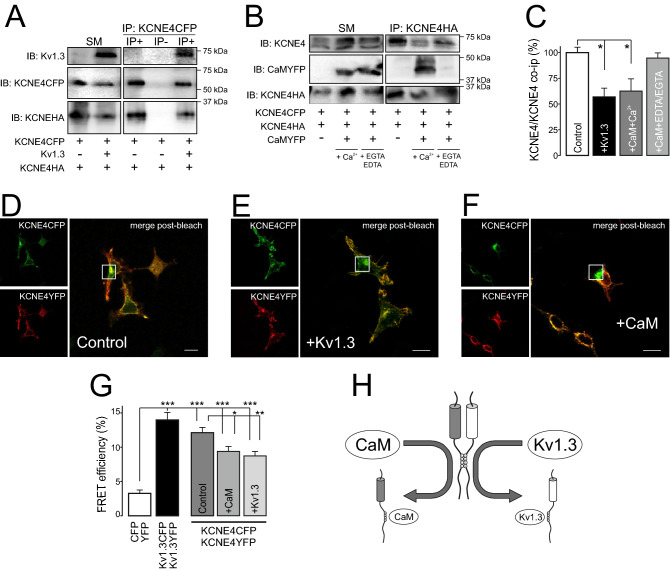
The presence of CaM and Kv1.3 impairs the dimerization of KCNE4. Coimmunoprecipitation of KCNE4-CFP and KCNE4-HA in the presence ( +) or absence (-) of Kv1.3 (**A**) and CaM (**B**). The experiment in the presence of CaM (+ CaM) was performed in the absence (+ 2 mM EDTA/2 mM EGTA) or the presence (+ Ca^2^^+^) of 2 mM Ca^2^^+^. KCNE4 was immunoprecipitated (IP) with select anti-tag antibodies (anti-GFP and anti-HA), and membranes were blotted (IB) against Kv1.3, KCNE4 and CaM with the indicated primary antibodies (anti-Kv1.3, anti-KCNE4, anti-GFP or anti-HA). Representative cropped blots are clearly separated by vertical white lines. (**C**) Relative KCNE4/KCNE4 coimmunoprecipitation in the presence of Kv1.3 and CaM. Control represents KCNE4/KCNE4 with no additions. The values represent the mean of 4 independent experiments. *p < 0.05 vs control (KCNE4-CFP/KCNE4-HA in the absence of further additions, Student’s *t*-test). (**D–F**) Representative FRET experiments between KCNE4YFP and KCNE4CFP in the absence (D, control) or presence of + Kv1.3 (**E**) or + CaM (**F**). Green panels, KCNE4-CFP (donor); red panels, KCNE4-YFP (acceptor); merged postbleach panels; yellow indicates colocalization; white square highlights the acceptor photobleached ROI analyzed. Bars represent 10 μm. (**G**) FRET efficiency quantification of KCNE4-CFP/KCNE4-YFP in the presence of CaM and Kv1.3. The values represent the mean of 20–30 cells. *p < 0.05; **p < 0.01; ***p < 0.001 (Student’s *t*-test). Control, no additions; + CaM, presence of CaM; + Kv1.3, presence of Kv1.3. CFP/YFP, negative controls; Kv1.3YFP/Kv1.3CFP, positive controls. (**H**) Schematic showing the interfering associations impairing KCNE4 dimer formation due to multiple tetraleucine motif interactions. The presence of either CaM or Kv1.3 would disrupt KCNE4 association, inhibiting dimer formation.

Our data indicated that the tetraleucine motif of KCNE4 is a hub for protein–protein interactions governing the dimerization of this peptide. In addition, this cluster is involved in Kv1.3 and CaM associations^20^. Both Kv and CaM interactions with KCNE4 are important for the physiological function of Kv channels^15^. Therefore, we wanted to analyze whether KCNE4 dimerization has a competitive effect fine-tuning the association of KCNE4 with CaM and Kv1.3. KCNE4 coimmunoprecipitation studies in the presence of either Kv1.3 (Fig. 6A) or CaM (Fig. 6B) indicated that the dimerization of KCNE4 is altered by both proteins (Fig. 6C). Thus, the presence of Kv1.3 decreased the KCNE4 dimer by one-half. In addition, Ca^2+^ is essential for KCNE4-dependent CaM-related Kv7.1 and Kv1.3 regulation^15,20^. Thus, CaM inhibited the dimerization of KCNE4 in the presence but not in the absence (EDTA/EGTA) of Ca^2+^ (Fig. 6C). Furthermore, FRET data, which indicated the molecular proximity between KCNE4-CFP/KCNE4-YFP, further supported that the dimerization of KCNE4 was also displaced by the presence of either Kv1.3 or CaM (Fig. 6D–G).

Keeping all these results and the previous evidence in mind, we postulated a model (Fig. 6H). KCNE4 dimerizes via its juxtamembrane C-terminal tetraleucine motif. In addition, this region is involved in the Kv1.3 association and CaM interaction. The dimerization of KCNE4 would balance oligomeric interactions fine-tuning the KCNE4 physiological effects. Therefore, the association with Kv1.3 and CaM would be in competition with the KCNE4 dyad formation.

**Figure 7:**
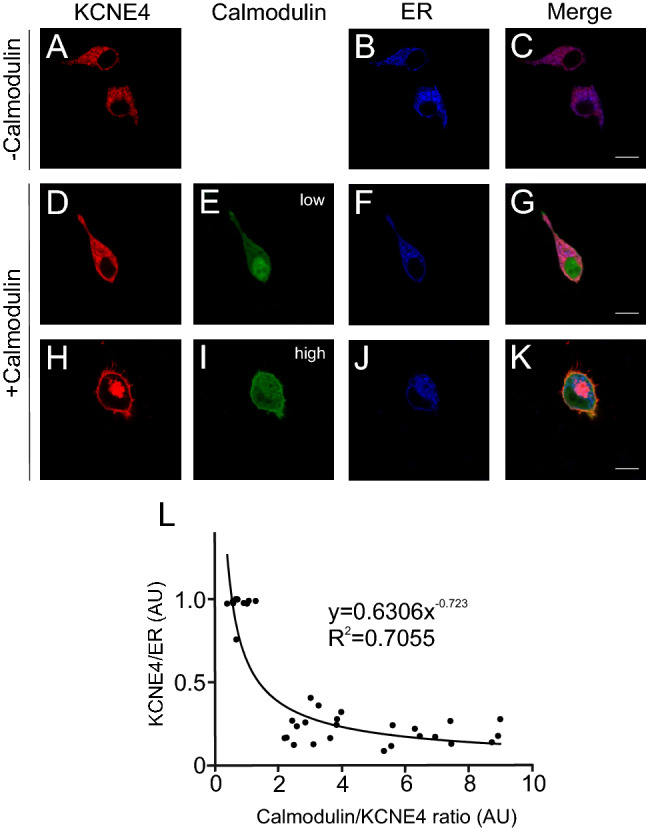
Endoplasmic reticulum colocalization of KCNE4 in the presence of CaM. HEK-293 cells were cotransfected with KCNE4-CFP, CaM-YFP and pDsRed-ER. (**A–C**) Colocalization of KCNE4 to the ER in the absence of CaM. (**D–K**) Colocalization of KCNE4 to the ER in the presence of CaM. (**D**–**G**) Representative images of a cell expressing low levels of CaM. (**H–K**) Representative images of a cell expressing high levels of CaM. (**A**, **D**, **H**) KCNE4-CFP, red; (**E**, **I**) CaM-YFP in green; (**B**, **F**, **J**) ER, blue; (**C**, **G**, **K**) merged panels. Purple indicates colocalization of KCNE4 and ER markers. Yellow, colocalization of KCNE4 and CaM. White and light pink indicate triple colocalization. Scale bars represented 10 µm. (**L**) ER colocalization of KCNE4 plotted against the CaM/KCNE4 ratio. All images were captured with the same laser intensity and photomultiplication parameters. A pixel-by-pixel analysis was performed to determine the relative KCNE4/ER and the CaM/KCNE4 ratios. The regression curve and a high R^2^ value are indicated. Values were from 20–30 cells. Note: High levels of CaM correspond to low ER colocalization of KCNE4.

Recent evidence indicates that increasing amounts of KCNE4 steadily decrease Kv1.3 abundance at the cell surface^30^. Furthermore, CaM facilitates the membrane expression of Kv7 channels^22^. CaM also competes with Kv1.3 for KCNE4 tetraleucine motif association, facilitating Kv1.3 escape from KCNE4-dependent ER retention. We wondered whether CaM, impairing KCNE4 dimerization, affects the cellular distribution of KCNE4. The ER colocalization of KCNE4 in the absence (Fig. 7A–C) or presence of variable (low, Fig. 7D–G; high, Fig. 7H–K) CaM expression was analyzed. All images were captured using the same parameters of laser intensity and photomultiplication. Therefore, a pixel-by-pixel analysis of both CaM and KCNE4 intensities was performed, and the data were plotted against the ER localization of KCNE4. The data fitted with an exponential decay showed a strong correlation. Therefore, we found that increasing amounts of CaM triggered a notable decrease in KCNE4 ER localization concomitant with an increase in the cell surface staining of KCNE4 (Fig. 7H–L).

**Figure 8:**
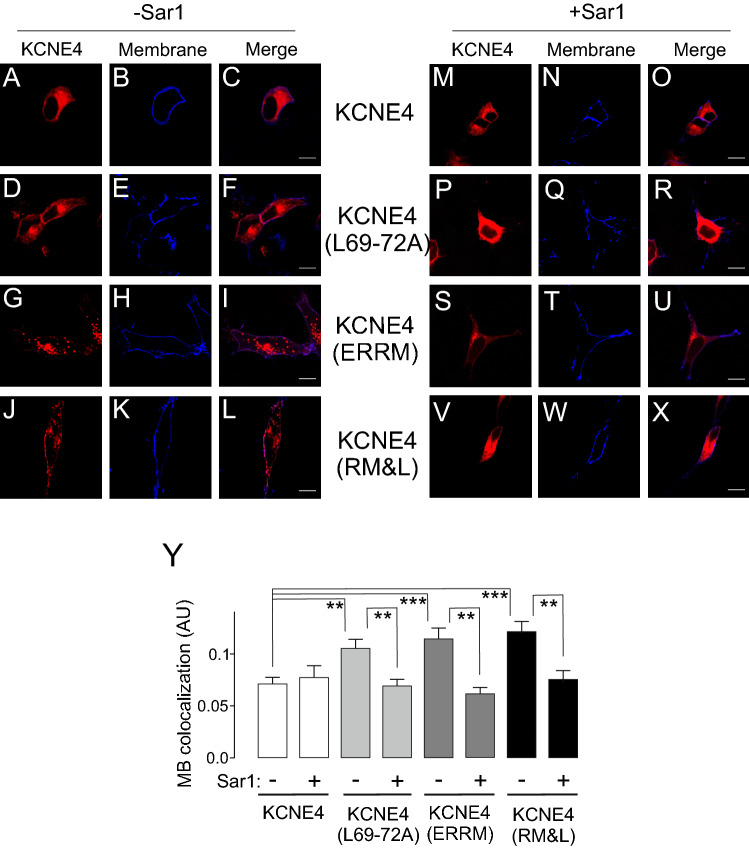
KCNE4 traffics to the plasma membrane via* a COPII-dependent mechanism.* HEK-293 cells were cotransfected with KCNE4WT and mutants (KCNE4(L69-72A), KCNE4(ERRM) and KCNE4(RM&L)) and the Akt-PH-pDsRed membrane marker in the absence (-Sar1, A-L) or the presence (+ Sar1, M-X) of HA-Sar1H79G. (A-C and M–O) KCNE4WT colocalization with the membrane marker. (D-F and P-R) KCNE4(L69-72A) colocalization with the membrane marker. (G-I and S-U) KCNE4(ERRM) colocalization with the membrane marker. (J-L and V-X) KCNE4(RM&L) membrane colocalization. Red panels, KCNE4. Blue panels, stained membrane. In the merged panels, purple indicates colocalization. Scale bars represent 10 µm. (Y) Quantification of membrane (MB) colocalization of KCNE4 in the absence ( −) or presence ( +) of HA-Sar1H79G (Sar1) using Mander’s coefficient. White columns, KCNE4 WT; light gray, KCNE4(L69-72A); dark gray, KCNE4(ERRM); black columns, KCNE4(RM&L). The values represent the mean of 20–30 cells. **p < 0.01; ***p < 0.001 One-way ANOVA and post hoc Tukey’s test.

Our data indicated that CaM facilitated the targeting of KCNE4 to the plasma membrane. Thus, we wanted to identify the mechanism that promoted KCNE4 forward trafficking to the cell surface. Although KCNE peptides exhibit notable intracellular retention, KCNE1 reaches the cell surface via COPII-dependent machinery^27^. KCNE1 and KCNE4 behave similarly, but only the latter contains the tetraleucine signature that facilitates CaM-dependent membrane surface expression. Therefore, we analyzed whether the COPII machinery mediated KCNE4 membrane targeting (Fig. 8). KCNE4 exhibited intracellular retention and minor membrane staining (Fig. 8A–C). The coexpression of Sar1 (H79G), which disrupts ER-to-Golgi trafficking^27^, did not alter the amount of KCNE4 localized at the membrane (Fig. 8A–C, Y). KCNE4(L69-72A) showed improved membrane targeting, similar to that obtained with KCNE4(ERRM), which has the ER retention motif disrupted, and KCNE4(RM&L), with both the ERRM and the tetraleucine signals altered (Fig. 8D–X). Interestingly, Sar1(H79G) clearly prevented membrane targeting under all conditions (Fig. 8Y). Therefore, our data suggest that, in addition to the tetraleucine motif, ERRM cooperates in the ER retention of KCNE4. In addition, we show that KCNE4, escaping the ER, is routed via conventional COPII-dependent anterograde trafficking to the cell plasma membrane.

## Discussion

The voltage-gated potassium channel Kv1.3 is crucial in the physiology of the immune system. The channel controls the membrane potential, driving Ca^++^ entry during the immune response. The abundance of Kv1.3 at the cell surface is in accordance with the magnitude of the cellular reaction^35^. KCNE4 is a regulatory subunit that controls the expression of Kv1.3 in the plasma membrane, fine-tuning this response^30^. Because KCNE4 is important for controlling channel functionality, its mechanism of action and architecture are of physiological relevance. Here, we demonstrate that, unlike other KCNE peptides, KCNE4 forms dimers. Dimerization is mediated by the tetraleucine motif in the C-terminal domain. Interestingly, this unique structural element creates a platform where protein-to-protein interactions take place. Thus, the capacities for KCNE4 interaction with Kv1.3 and CaM with and KCNE4 dimerization are determined at this motif. CaM-dependent dimerization of KCNE4 tunes KCNE4 ER retention and the KCNE4-Kv1.3 association. Dimerization provides additional ERRM to the complex that balances the CaM association. Furthermore, the evidence indicates that the forward trafficking of KCNE4 to reach the membrane surface is realized through the COPII-dependent machinery. Our data are of physiological relevance because the dimerization attenuates the effects of the KCNE4 regulatory subunit on Kv1.3. In addition, the KCNE4 interaction with CaM, which hides the tetraleucine motif, situates this important signaling molecule to balance Kv1.3 physiological functions.

The tetraleucine motif at the juxtamembrane domain of the KCNE4 C-terminus is a structural platform where essential proteins for Kv1.3-dependent physiology interface. Thus far, this KCNE4 feature is unique within the KCNE family. Dileucine motifs promote endocytosis, protein–protein interactions or basolateral trafficking^36–39^. In this context, leucine-enriched domains mediate the oligomerization of Toll-like receptors^40^. However, leucine residues are mainly located at every third amino acid position in these domains^41^. The KCNE4 tetraleucine signature forms a coil-like domain that can be transferred to other KCNE peptides. Moreover, our data demonstrated that this mechanism acts as a trafficking modulator. Mutation of this cluster, as well as the ERRM, facilitates plasma membrane targeting of KCNE4. Dimerization provides redundant signals to the KCNE4 dyad, retaining a pool of peptides at the ER. Thus, not only the interaction with Kv1.3, but also with CaM, is controlled^20^. Upon association with CaM or Kv1.3, KCNE4 units may travel either with CaM to the membrane or, by masking the forward traffic motifs of Kv1.3, retain the channel within in the cell, thereby tuning the channel expression at the cell surface. Both mechanisms reveal as important players controlling Kv1.3-asociated cellular responses^42,43^.

Evidence links dileucine clusters with protein trafficking. For instance, the ClC-2 chloride channel, which might compensate for the malfunction of CFTR in absorptive epithelia during cystic fibrosis, contains a dileucine motif. Mutations of this signature alter the basolateral targeting of the channel^44^. Similarly, the dileucine motif of human organic solute transporter beta plays a critical role in its association with the alpha subunit and cell surface polarization^37^. However, similar to that of KCNE4, other evidence raises an alternative possibility. The acid-sensing ion channel ASIC2a has a double dileucine motif (LLDLL) in the C-terminal juxtamembrane region. Altering the LLDLL sequence, either full or in part, increases the surface levels of the channel^45^. Moreover, mutation of the carboxyl terminal dileucine sequence of the LHβ subunit increases trafficking and exocytosis^46^. In this context, our data indicated that disrupting or masking the tetraleucine motif hinders dimerization, thereby facilitating membrane targeting of KCNE4. Therefore, in a KCNE4 monomer, although it contains an ERRM, the strength of the retention signal may be counterbalanced by associating with CaM. This mechanism is in agreement with the Ca^2+^/CaM-dependent regulation of KCNE4 of Kv channels, such as Kv7.1 or Kv1.3. CaM, upon calcium stimuli, can interact with KCNE4 at the tetraleucine motif^15,20^. Therefore, CaM fine-tunes the dimerization and promotes KCNE4 anterograde trafficking through a COPII-dependent mechanism. Thus, Ca^2+^ signaling controls the final fate of KCNE4, which in turn modulates Kv1.3 function, which is profoundly related to the Ca^2+^ trigger in leukocytes. The precise balance between several interactions in a unique cluster, masking the interaction sites to prevent competing associations, fine-tunes the final function of a multitask channel such as Kv1.3^16^. A similar cloaking mechanism is evident for E-cadherin. A dileucine motif in the juxtamembrane cytoplasmic domain is required for E-cadherin endocytosis. When mutated, this protein cannot be internalized. P-120, an E-cadherin interactor, associates and masks this motif, inhibiting endocytosis^47^. Furthermore, our data indicate that KCNE4 contains routing information to the plasma membrane via COPII-dependent mechanisms. KCNEs, which contain strong ERRM signals at their C-terminus, share a notable intracellular retention capacity. Surprisingly, without canonical sequences, the COPII-dependent mechanism is a major characteristic of KCNE peptides because, similar to KCNE4, KCNE1 uses this route^27^. In this sense, N-glycosylation facilitates KCNE anterograde trafficking through the secretory pathway, but the specific mechanisms have not been dissected^48^. The balance between forward trafficking and retention signaling is highly skewed toward the latter in KCNEs. Regardless of whether the assembly of KCNE and Kv channels takes place at the cell surface or through the secretory pathway, CaM controls cell surface channels by modulating KCNE4 trafficking^15,27,49^.

We demonstrated that Kv1.3, KCNE4 and CaM are closely related because they interact via the tetraleucine cluster in KCNE4. A balanced competition warrants no tripartite complexes^20^. Our data support the dimerization of KCNE4 as a regulating mechanism for such interactions and CaM displaced KCNE4 dyad formation. These data, similar to those of Kv7.1/KCNE1 and Kv7.1/KCNE3, further support that single monomeric forms of KCNE4 occupy the cleavage between subunits within the Kv tetrameric structure^20,28,29^. However, in contrast to Kv7.1, Kv1.3 does not need CaM to associate with KCNE4^15^. CaM indirectly interacts with Kv1.3 in a Ca^2+^-dependent manner, forming a multiprotein complex with CaM kinase II (CaMKII)^50,51^. Although the regulation of Kv1.3 by Ca^2+^/CaM in leukocytes is under debate, CaM-dependent KCNE4 dimerization situates this important signaling molecule in the equation. Ca^2+^- and CaM-dependent signaling pathways are key to T cell activation. The immunological synapses enriched in lipid rafts concentrate Ca^2+^-dependent targets. KCNE4, retaining Kv1.3 at the ER, impairs the localization of Kv1.3 to lipid rafts. Therefore, our data are of physiological relevance because indicates that the KCNE4 tetraleucine motif is an associative platform in which different targets may compete for signaling interactions fine-tuning the cell physiology.

## Methods

### Expression plasmids, chimeric channels and site-directed mutagenesis

The rKv1.3 in pRcCMV, provided by T.C. Holmes (University of California, Irvine), was subcloned into pEYFP-C1, pECFP-C1 and pEGFP-C1 (Clontech). hKv1.5 has been widely analyzed^26^. Mouse KCNE4 in pSGEM was obtained from M. Sanguinetti (University of Utah, Salt Lake City, UT). mKCNE4 was introduced into pEYFP-N1, pECFP-N1 and pEGFP-N1 (Clontech). hKCNE1-HA and hKCNE2-HA, obtained from S. de la Luna (CRG, Barcelona, Spain), were introduced into pECFP-N1, pEYFP-N1 and pEGFP-N1 (Clontech). CaMYFP was obtained from A. Villarroel (Universidad País Vasco-CSIC). An endoplasmic reticulum marker (pDsRed-ER), soluble cyan fluorescent protein (pECFP-N1), green fluorescent protein (pEGFP-N1) and yellow fluorescent protein (pEYFP-N1) were obtained from Clontech. The plasma membrane marker Akt-PH-pDsRed (pDsRed-tagged pleckstrin homology (PH) domain of Akt) was a kind gift from F. Viana (Universidad Miguel Hernández, Spain). The dominant-negative GTPase mutant HA-Sar1H79G was provided by R. Pepperkok (EMBL, Germany)^19–21^.

For counting the bleaching steps in nonmoving spots, a loopBAD-tag was added. The loop-BAD tag was introduced into GFP-Kv1.3, KCNE1-GFP and KCNE4-GFP at NruI restriction sites. The Kv2.1-loopBAD-YFP and BirA coding plasmids were previously described^30,52^.

The KCNE4-CFP tetraleucine motif (L69–72) was mutated to alanine by single-site–directed mutagenesis using a QuikChange multisite-directed mutagenesis kit (Agilent Technologies), generating the KCNE4(L69–72A)-CFP mutant. A tetraleucine motif similar to that in KCNE4 was incorporated into KCNE2-CFP (KCNE2(L83–86)-CFP). KCNE4 (ERRM), with ERRM disruption, and KCNE4 (RM&L), containing both modified ERRM and tetraleucine motifs, have been fully characterized previously^20^. All constructs and mutants were verified by automated DNA sequencing.

### Cell culture and transient transfection

HEK-293 cells (Sigma-Aldrich) were cultured in DMEM (LONZA) containing 10% fetal bovine serum (FBS) supplemented with penicillin (10,000 U/ml), streptomycin (100 μg/ml) (Gibco), 4.5 g/l glucose and L-glutamine (4 mM).

For confocal imaging and coimmunoprecipitation experiments, cells were seeded (70–80% confluence) in either 6-well dishes containing poly-D-lysine-coated coverslips or 100-mm dishes. Metafectene PRO (Biontex) was used for transfection according to the supplier’s instructions. The amount of transfected DNA was 4 μg for a 100-mm dish and 500 ng for each well of a 6-well dish. Next, 4–6 h after transfection, the mixture was removed from the dishes and replaced with fresh culture medium. All experiments were performed 24 h after transfection^20,21^.

For patch-clamp experiments, trypsinized cells from a confluent culture in a 100-mm dish were electroporated with 1 μg of DNA using a Bio-Rad Gene Pulser Xcell system with a 0.2-cm gap cuvette and a single 110-V 25-ms pulse^30^.

For total internal reflection fluorescence (TIRF) microscopy experiments, trypsinized cells from a confluent culture in a 100-mm dish were electroporated with 25–100 ng of the desired DNA plus 100 ng of BirA DNA (encoding a biotin ligase to biotinylate the loopBAD-tagged proteins) using a Bio-Rad Gene Pulser Xcell system, as previously described. Transfected cells were plated on 35-mm glass-bottom dishes (MatTek) previously coated with collagen and EZ-Link NHS-PEG12-Biotin (Pierce, Thermo Scientific). The next day, TIRF experiments were performed after incubation of the cells with neutravidin (50 nM) to immobilize channels onto glass as previously described^20,35^.

### Protein extraction, coimmunoprecipitation and western blotting

HEK-293 cells were washed twice in cold PBS and lysed on ice with a lysis solution (1% Triton X-100, 10% glycerol, 50 mM HEPES, 150 mM NaCl at pH 7.2) supplemented with protease inhibitors (1 µg/ml aprotinin, 1 µg/ml leupeptin, 1 µg/ml pepstatin and 1 mM phenylmethylsulfonyl fluoride). Lysates were gently mixed for 10 min and spun (10 min at 12,000 × g). The supernatant was transferred to a new tube, and the protein contents were determined by using a Bio-Rad protein assay^20,21^.

For coimmunoprecipitation, 1 mg of protein added to lysis buffer at a final concentration of 500 µl and used for immunoprecipitation (150 mM NaCl, 50 mM HEPES, 1% Triton X-100 at pH 7.4) supplemented with protease inhibitors. Samples were precleared with 50 µl of protein A-Sepharose beads (GE Healthcare) for 1 h at 4 °C with gentle mixing. Next, each sample was incubated in a small chromatography column (Bio-Rad Micro Bio-Spin™ chromatography columns), which contained 2.5 µg of anti-GFP antibody previously cross-linked to protein A-Sepharose beads, for 2 h at room temperature (RT) with gentle mixing. The columns were centrifuged for 30 s at 1,000 × g. Supernatants were stored at -20 °C. The columns were washed four times with 500 µl of lysis buffer and centrifuged for 30 s at 1,000 × g. Finally, the columns were incubated with 100 µl of 0.2 M glycine (pH 2.5) and spun for 30 s at 1,000 × g for elution^19–21^.

Irreversible cross-linking of the antibody to the Sepharose beads was performed after incubation of the antibody with protein A-Sepharose beads for 1 h at RT. The beads were then incubated with 500 µl of 5.2 mg/ml dimethyl pimelimidate (Pierce) for 30 min at RT with gentle mixing. The columns were then washed four times with 500 µl of 1 × TBS, four times with 500 µl of 0.2 M glycine (pH 2.5) and three more times with 1 × TBS (0.1% Triton X-100, 10% glycerol, 150 mM NaCl, 50 mM HEPES at pH 7.4). Finally, the columns were incubated with protein lysates to perform immunoprecipitation as described above.

For the coimmunoprecipitation experiments in the presence of CaM, the samples were precleared for 1 h at 4 °C by mixing samples with 50 µl of protein A-Sepharose beads (GE Healthcare) previously washed with 1 × TBS and centrifuged for 30 s at 5,000 × g. Next, supernatants were incubated for 2 h at RT with 8 ng of anti-HA antibody (Proteintech) previously cross-linked to Protein A-Sepharose beads. The samples were centrifuged (30 s, 5.000 × g), and the pellet was washed 3 times with 1 × TBS buffer. Finally, proteins were eluted upon the addition of 20 µl of SDS Laemmli loading buffer (5x) and 80 µl of H_2_O and boiled for 7 min. Elutions were briefly centrifuged, and the supernatans were prepared for western blotting^19–21^.

All experimental protocols were approved by the ethical committee of the Universitat de Barcelona in accordance with the European Community Council Directive 86/609 EEC. We also confirm that all experiments were carried out in compliance with the ARRIVE guidelines (https://arriveguidelines.org). Rats were briefly anesthetized with isoflurane, and tissues were extracted immediately after euthanasia. Tissues were homogenized in RIPA lysis buffer (1% Triton X-100, 1% sodium deoxycholate, 0.1% SDS, 50 mM Tris–HCl pH 8.0, 150 mM NaCl) supplemented with protease inhibitors. Total lysates were spun for 10 min at 10,000 × g to remove debris. Supernatants were used to analyze protein expression by western blot.

Protein samples (50 μg), supernatants, immunoprecipitates and total tissue lysates were boiled in Laemmli SDS loading buffer and separated by 10–15% SDS-PAGE. Next, the proteins were transferred to PVDF membranes (Immobilon-P; Millipore) and blocked in 0.2% Tween-20-PBS supplemented with 5% dry milk before immunoreaction with antibodies. Filters were immunoblotted with antibodies against Kv1.3 (1/500, NeuroMab), HA (1/1,000, Sigma), KCNE4 (1/800, Proteintech), GFP (1/1,000, Roche) or calmodulin (1/600, Millipore). Finally, the membranes were washed with 0.05% Tween 20 PBS and incubated with horseradish peroxidase-conjugated secondary antibodies (Bio-Rad).

The dimerization of KCNE4 WT-CFP and KCNE4 (L69–72A)-CFP was analyzed in non-denaturing conditions. Briefly, total lysates were mixed with sample buffer (50 mM Tris–HCl pH 6.8, 10% glycerol, 0.2% bromophenol blue) and loaded in a non-denaturizing SDS-PAGE (5% acrylamide/bis-acrylamide (30%-0.8% w/v), 0.29 M Tris–HCl pH 8.8, 0.1% SDS, 0.1%). Western Blot was performed as abovementioned and PVDF membranes were incubated with anti-GFP (1/1,000, Roche) and horseradish peroxidase-conjugated secondary antibodies (Bio-Rad).

### DMP cross-linking protocol

Twenty-four hours after transfection, HEK-293 cells were washed twice in cold PBS and lysed on ice with lysis buffer (50 mM boric acid, 100 mM K acetate, 2 mM MgCl_2_, 1 mM EGTA and 1% Triton X-100; pH 8.5). One-half of the proteins was incubated with 5 mM DMP (dimethyl pimelimidate) for 1 h, while the other half was maintained for use as controls. The reaction was stopped by adding 1 ml of 0.5 M (pH 6.8). Protein samples (50 μg) were boiled in Laemmli SDS loading buffer and separated on 7% SDS-PAGE gels.

### Electrophysiology

HEK-293 cells were trypsinized 24 h after transfection and plated on 35-mm glass-bottom dishes coated with Matrigel^30^. After 2–4 h, the cells were washed extensively with whole-cell external recording solution containing (in mM) 150 NaCl, 5 KCl, 10 CaCl_2_, 2 MgCl_2_, 10 glucose, and 10 HEPES at pH 7.4. Whole-cell currents were recorded using the patch-clamp technique in the whole-cell configuration with a HEKA EPC10 USB amplifier (HEKA Elektronik). PatchMaster software (HEKA) was used for data acquisition. We applied a stimulation frequency of 50 kHz and a filter at 10 kHz. The capacitance and series resistance compensation were optimized. In most experiments, we obtained 80% compensation of the effective access resistance. Micropipettes were prepared from borosilicate glass capillaries (Harvard Apparatus) using a P-97 puller (Sutter Instrument) and fire polished. The pipettes had a resistance of 2–4 MΩ when filled with a solution containing (in mM): 4 NaCl, 150 KCl, 1 MgCl_2_, 0.5 EGTA, 5 K_2_ATP, and 10 HEPES at pH 7.4. Cells were clamped at a holding potential of − 80 mV. To evoke voltage-gated currents, cells were stimulated with 200 ms square pulses to + 60 mV. Data analysis was performed using FitMaster (HEKA) and SigmaPlot 10.0 software (Systat Software). All recordings were performed at room temperature (21–23 °C).

### KCNE4 complex modeling

KCNE4 was modeled using high-resolution templates of homologs available from the Protein Data Bank (http://www.rcsb.org/pdb). The N-terminus, transmembrane domain and proximal C-terminus (residues 1–98), which contains the tetraleucine motif, were modeled with KCNE1 (PDB ID: 2K21). The final molecular graphic representation was created using PyMOL v1.4.1 (http://pymorl.org/) as previously described^20^.

### Confocal microscopy, image analysis and FRET

For confocal image acquisition, cells were seeded on polylysine-coated coverslips and transfected 24 h later. The next day, the cells were quickly washed twice, fixed with 4% paraformaldehyde for 10 min, and washed three times for 5 min each time with PBS-K^+^. Finally, coverslips were added to microscope slides (ACEFESA) with house Mowiol mounting media. The coverslips were allowed to dry for at least one day before imaging. All procedures were performed at RT. All images were acquired with a Leica TCS SP2 AOBS microscope (Leica Microsystems) equipped with argon and helium–neon lasers. All experiments were performed with a 63 × oil-immersion objective lens NA 1.32. Colocalization analysis was performed with ImageJ software (NIH, Bethesda, MD, USA) as previously described. A pixel-by-pixel colocalization study using JACoP (Just Another Colocalization Plugin) was used. Mander’s overlap coefficients (MOC), which are proportional to the amount of fluorescence of the colocalizing pixels in each color channel, were obtained^19–21^. In some experiments the colocalization of KCNE4 with some subcellular markers was analyzed. HEK 293 cells were co-transfected with KCNE4 and the endoplasmic reticulum marker (ER, pDsRed-ER) and the plasma membrane marker (MB, Akt-PH-pDsRed). In addition, KCNE4 transfected cells were further processed for immunocytochemistry. Thus, after fixation, cells were further permeabilized with 0.1% Triton X-100 for 20 min. After 60 min of incubation with blocking solution (10% goat serum, 5% nonfat milk, and 0.05% Triton X-100), the cells were incubated with anti-GM130 (1:1000; BD Transduction Laboratories) and anti-TGN46 2F11 (1:100; bioNova científica) in 10% goat serum and 0.05% Triton X-100 overnight at 4 °C for cis-Golgi and trans-Golgi networks respectively. Next, the cells were incubated with Cy5-conjugated secondary antibodies for 2 h at RT and further mounted with Mowiol.

The fluorescence resonance energy transfer (FRET) via the acceptor photobleaching technique was measured in discrete ROIs (regions of interest). Fluorescent proteins from fixed cells were excited with the 458 nm or 514 nm beam lines using low excitation intensities. Next, 475 to 495 nm bandpass and > 530 nm longpass emission filters were applied. The YFP protein was bleached using maximum laser power. We obtained acceptor intensity bleaching of approximately 80%. Posteriorly, images of the donors and acceptors were taken. The FRET efficiency was calculated using the equation [(F_CFPafter_ – F_CFPbefore_)/F_CFPbefore_]*100, where F_CFPafter_ was the fluorescence of the donor after bleaching and F_CFPbefore_ was the fluorescence before bleaching. The loss of fluorescence as a result of scanning was corrected by measuring the CFP intensity in the unbleached part of the cell^20^.

### TIRF and bleaching steps of single fluorescent protein complexes

The single bleaching quantitative approach was first described by Isacoff and collaborators^31^ and is based on TIRF microscopy to visualize single GFP-tagged proteins on the cell surface, as previously described^20,35^. To immobilize proteins and analyze the number of bleaching steps, cells were transfected with loopBAD-tagged proteins in the presence of BirA, which encodes DNA for tag biotinylation. The cells seeded in a biotin-collagen-coated glass-bottom dish were incubated with neutravidin (50 nM) at 37 °C for 30 min before imaging. Neutravidin binds the cell surface biotinylated Kv1.3-, KCNE1- and KCNE4-loopBAD-GFP and, simultaneously, the biotinylated glass surface, fixing the cell surface proteins to monitor the fluorescently immobile GFP spots. The amount of DNA transfected was adjusted to achieve a low membrane density for imaging and counting. Multiple spots were imaged, but the density was low enough to minimize the probability of two channels lying within the same diffraction-limited spot. Transfected HEK-293 cells were imaged within 24 h after electroporation in HEK physiological saline buffer consisting of (in mM) 146 NaCl, 4.7 KCl, 2.5 CaCl_2_, 1 MgCl_2_, 10 glucose, and 10 HEPES at pH 7.4. Videos were processed and analyzed using Volocity software. The intensity of the 6 × 6 pixel ROIs was added and calculated for all durations of the videos. The intensity of the ROIs was plotted against time. The GFP bleaching steps were counted and statistically analyzed. GFP bleaching experiments were performed with a Nikon Eclipse Ti Perfect-Focus system equipped with a TIRF/wide-field fluorescence microscope and AOTF-controlled 405, 488, 561 nm diode lasers (100 mW each), and an Intensilight wide-field light source. A 100 × PlanApo TIRF, 1.49 NA, objective lens was used for image acquisition. Emissions were collected through a Sutter Lambda 10–3 filter wheel containing the appropriate bandpass filters. The microscope was equipped with an Andor iXonEMCCD DU-897 camera, 512 × 512. For TIRF image acquisition, an incident angle of 63.3° was used^30,35,52^.

### Statistics

The results are expressed as the means ± SE. Student’s *t*-test, one-way ANOVA and Tukey’s post hoc test and two-way ANOVA were used for statistical analysis (GraphPad PRISM v5.01). *P* < 0.05 was considered statistically significant.

## Supplementary Information

Supplementary Information.
