# Supplementary material for: Calmodulin-dependent KCNE4 dimerization controls membrane targeting
Source: Sci Rep. 2021 Jul 7;11:14046. doi: 10.1038/s41598-021-93562-5 (PMC8263776; doi:10.1038/s41598-021-93562-5)
Supplement: Supplementary file 1 — Supplementary Information. [file 41598_2021_93562_MOESM1_ESM.pdf]

Supplementary Information

Calmodulin-dependent KCNE4 dimerization controls membrane targeting

Sara R. Roig[1,2], Laura Solé[1,3], Silvia Cassinelli[1], Magalí Colomer-Molera[1], Daniel Sastre[1], Clara Serrano-Novillo[1], Antonio Serrano-Albarrás[1], M. Pilar Lillo[4], Michael M Tamkun[3], Antonio Felipe[1]

[1]Molecular Physiology Laboratory, Dpt. de Bioquímica i Biomedicina Molecular, Institut de Biomedicina (IBUB), Universitat de Barcelona, Avda. Diagonal 643, 08028 Barcelona, Spain. [2]Imaging Core Facility, Biozentrum, University of Basel, 4056 Basel, Switzerland; [3]Department of Biomedical Sciences, Colorado State University, Fort Collins, Colorado 80523. [4]Instituto de Química Física Rocasolano, CSIC, 28006 Madrid, Spain.

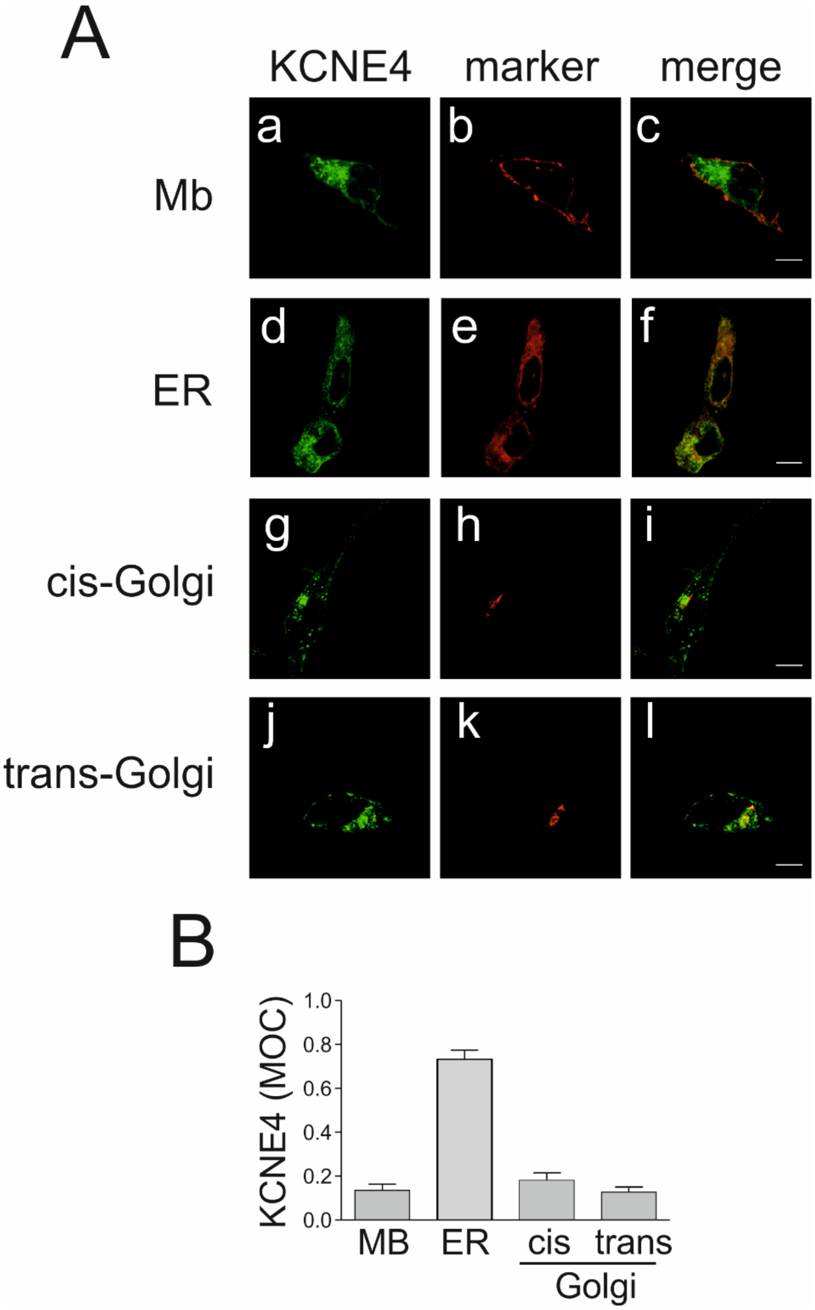

Supplementary Figure 1. *Subcellular localization of KCNE4.* Representative confocal images of HEK-293 cells transfected with KCNE4. (Aa-c) KCNE4-transfected cells were cotransfected with a plasma membrane marker (MB). (Ad-f) KCNE4-transfected HEK-293 cells were cotransfected with an endoplasmic reticulum marker (ER). (Ag-i) KCNE4-transfected cells were further stained against cis-Golgi network (GM130). (Aj-l) KCNE4-transfected HEK-293 cells were further stained against trans-Golgi (TGN46). Left panels, KCNE4 in green; center panels, cellular markers in red; right panels, merge in yellow. Bars represent 10 μm. (B) Histogram showing the colocalization between KCNE4 and the markers based on Mander's overlap coefficient (MOC). The values represent mean ± SE of >40 cells.

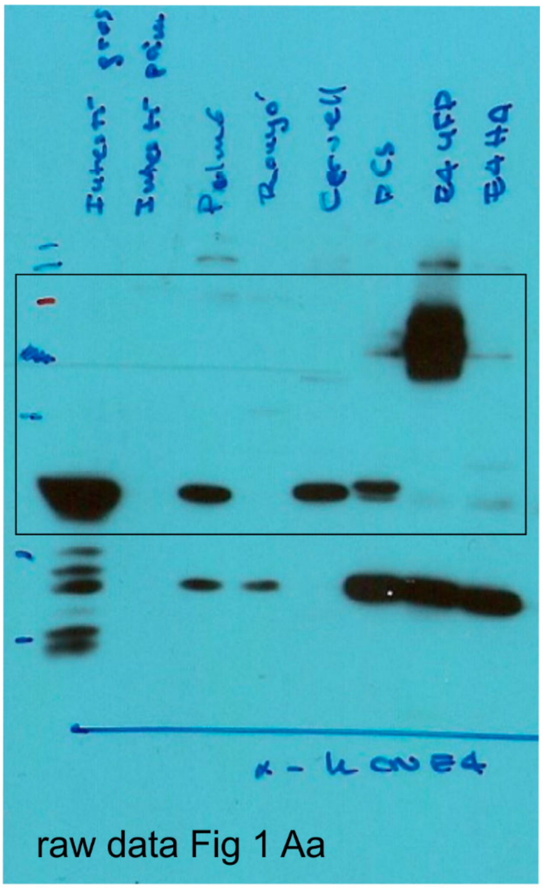

raw data Fig 1 Aa

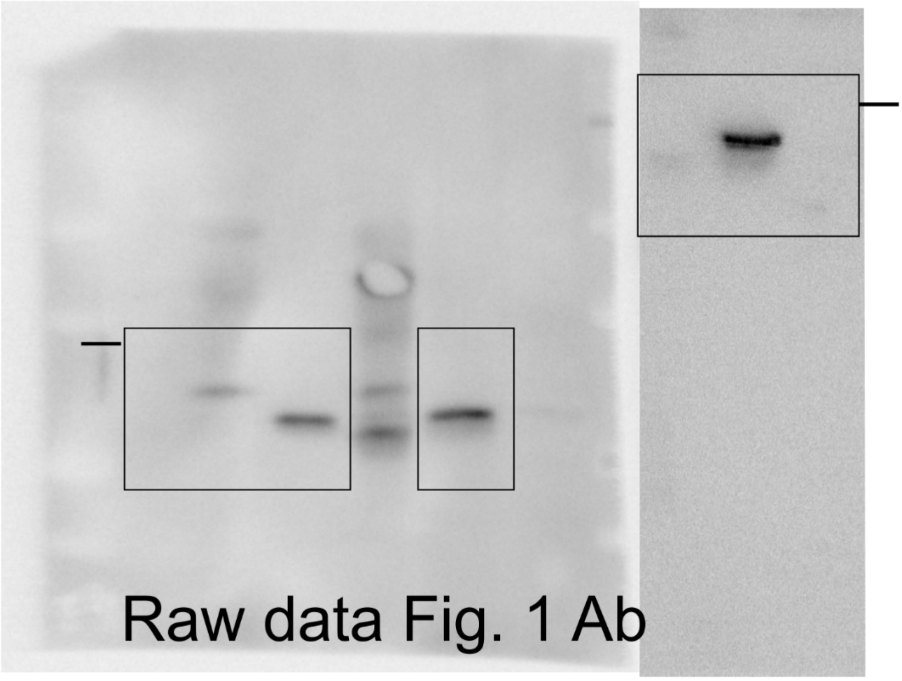

Raw data Fig. 1 Ab

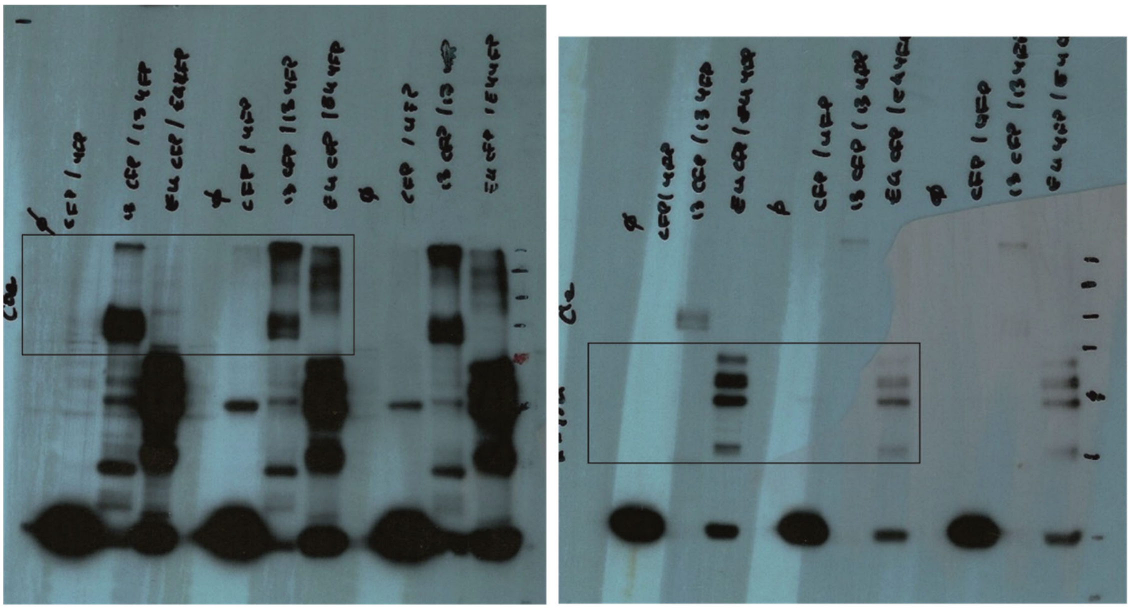

Raw data Fig 3 B

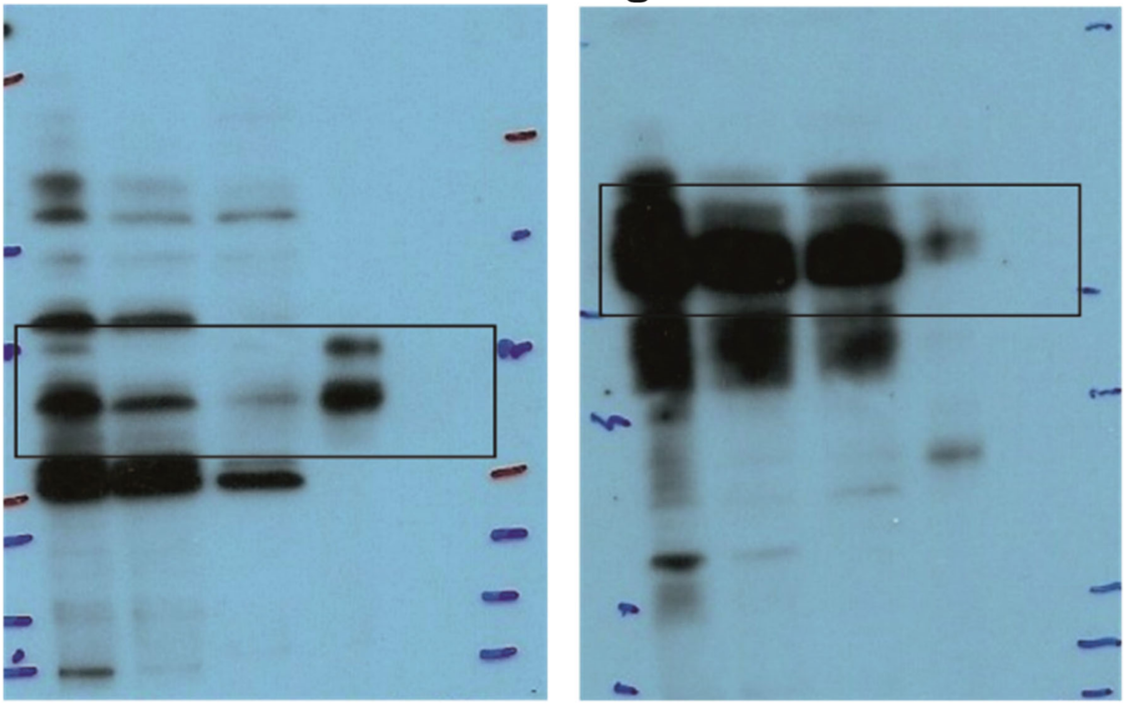

Raw data Fig 3 C

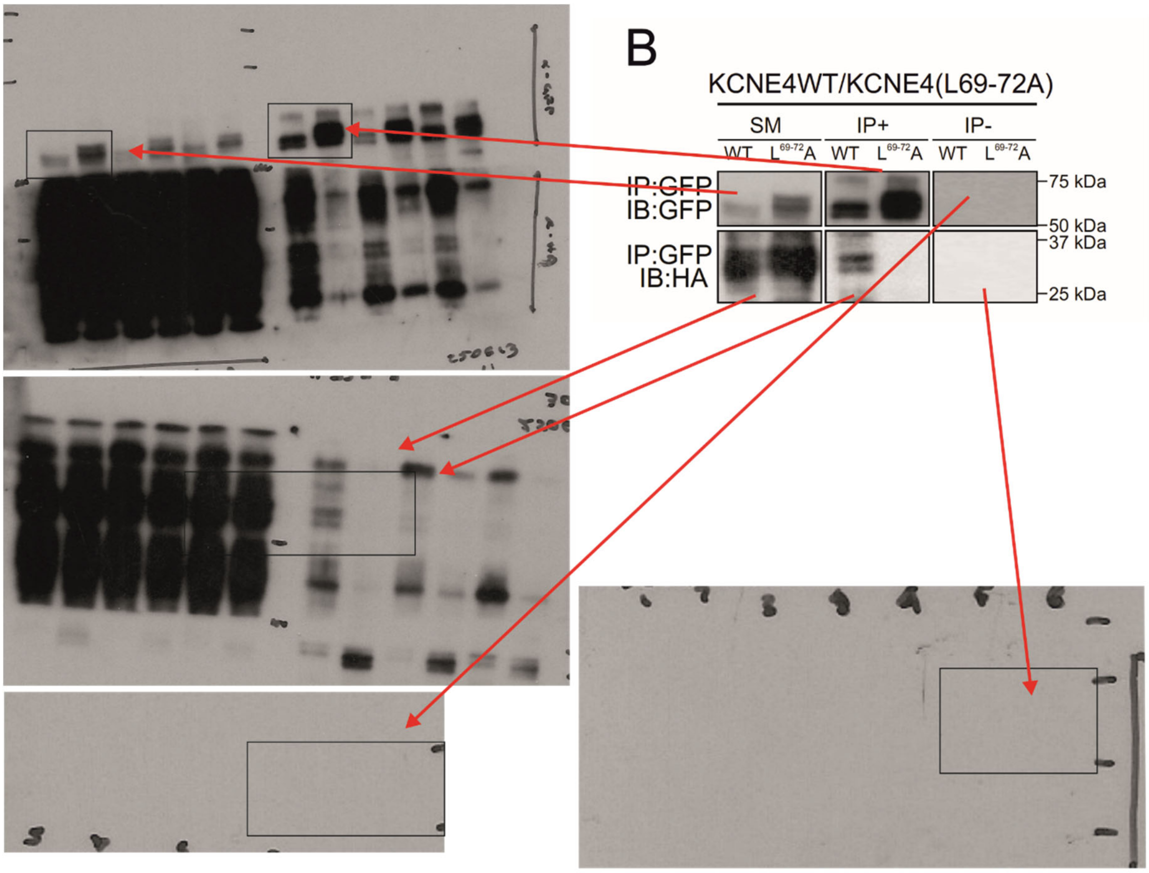

Raw data Fig 5B

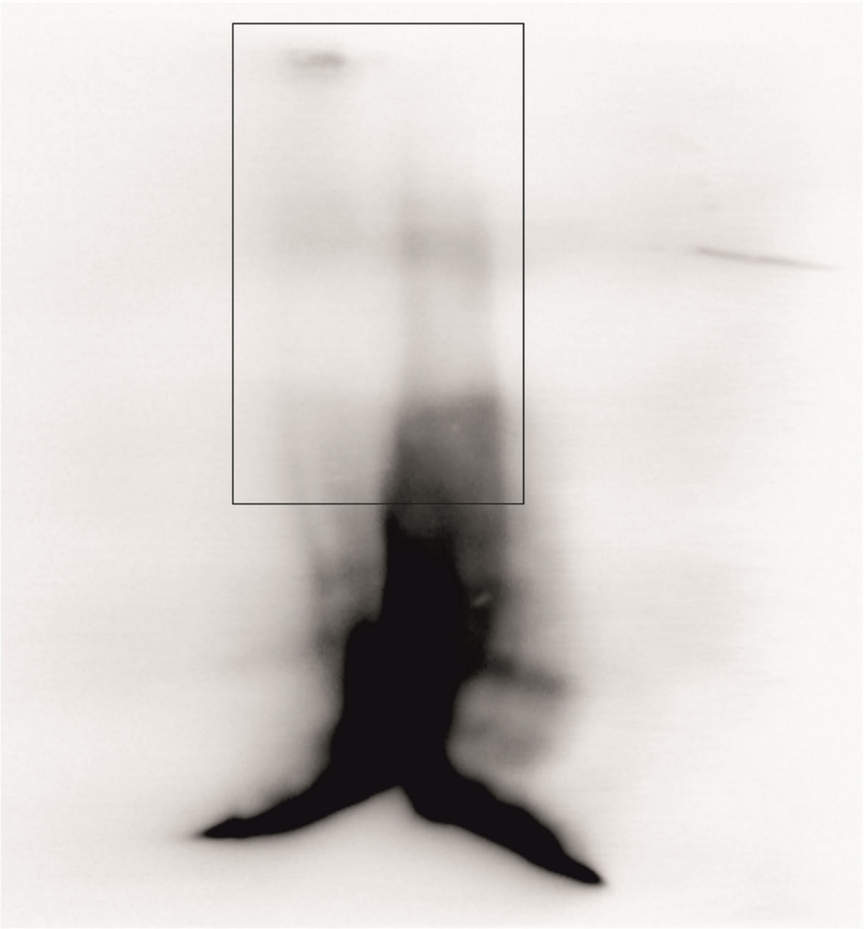

Raw data Fig 5C

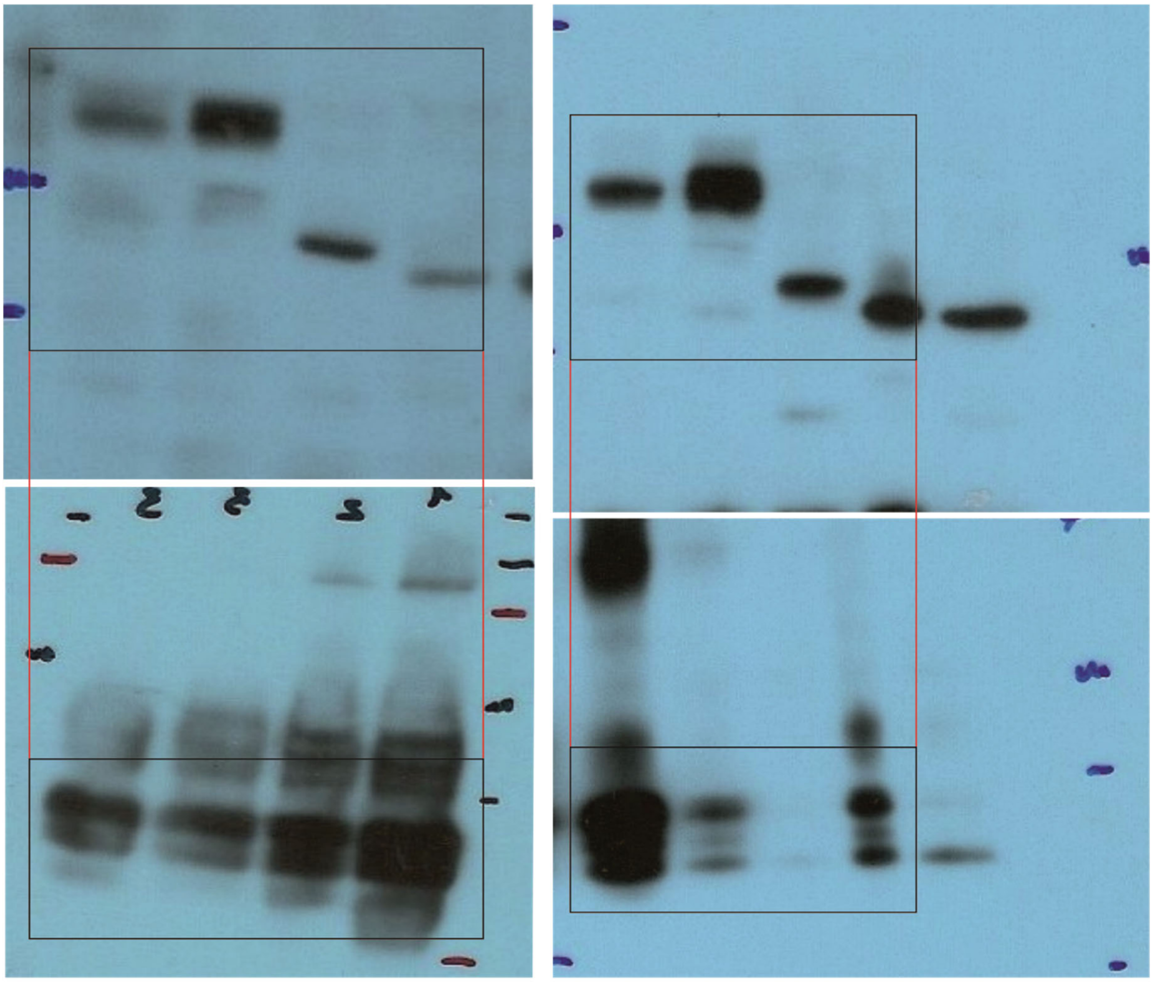

Raw data Fig 5E

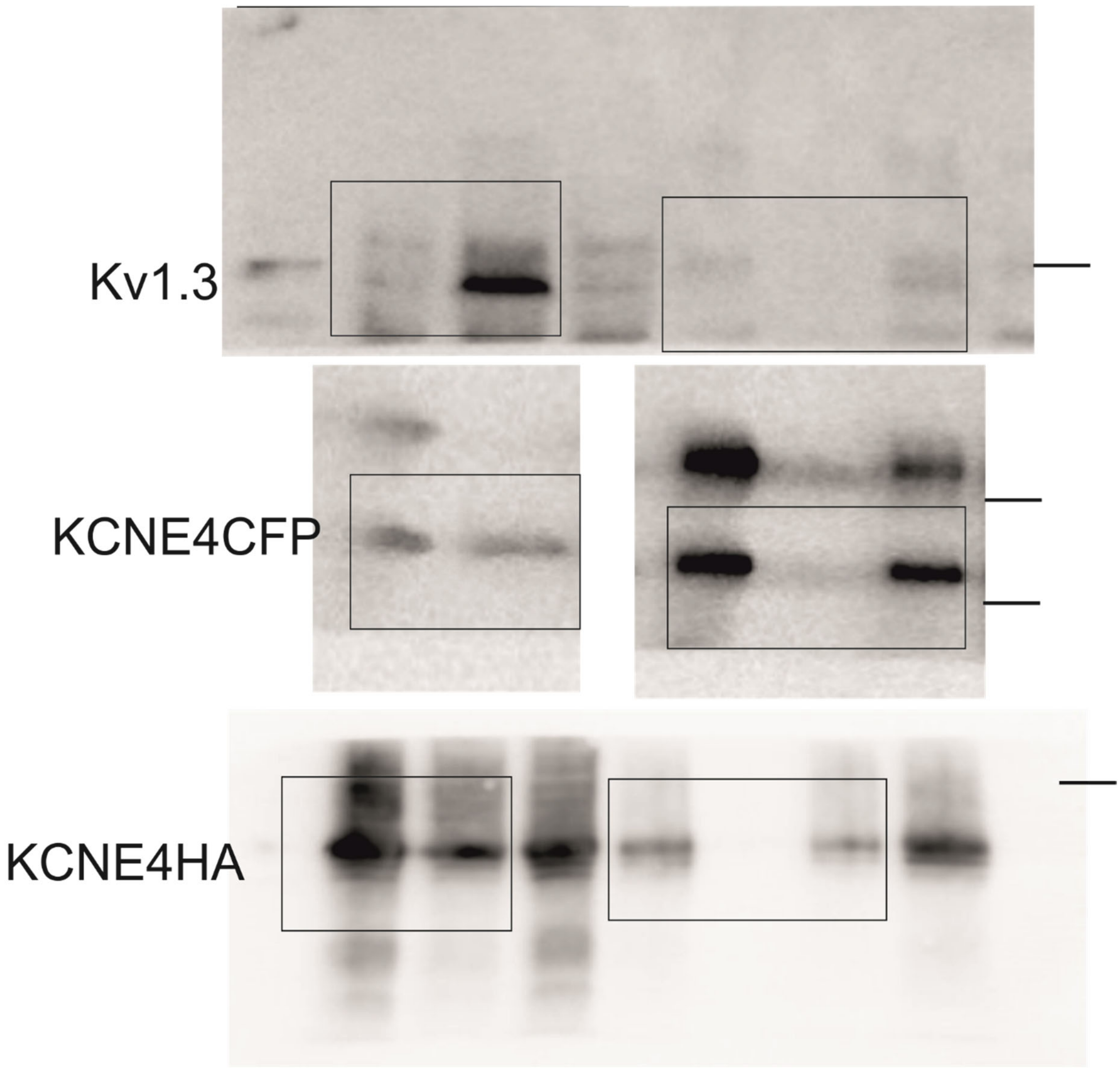

Raw data Fig 6A

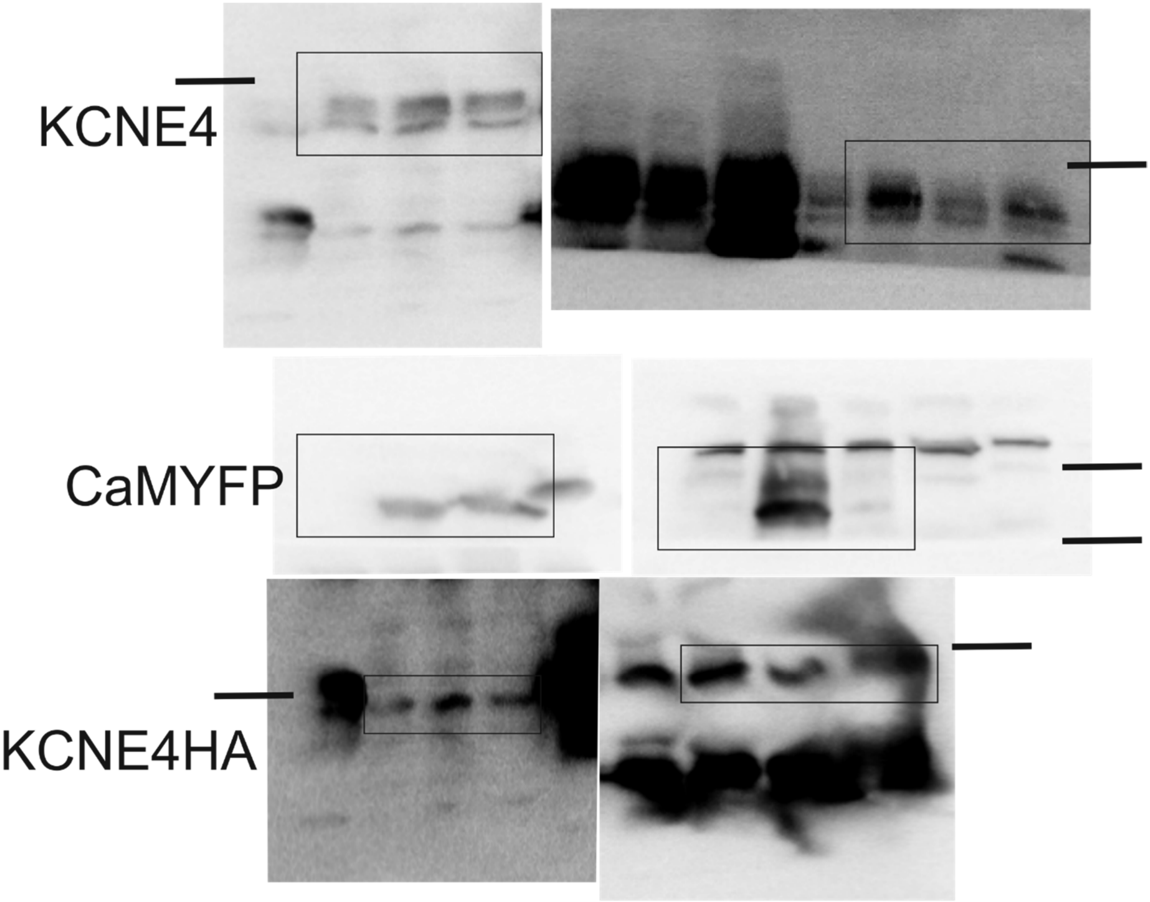

Raw data Fig. 6B
